# Artificial intelligence-enhanced retinal imaging as a biomarker for systemic diseases

**DOI:** 10.7150/thno.100786

**Published:** 2025-02-18

**Authors:** Jinyuan Wang, Ya Xing Wang, Dian Zeng, Zhuoting Zhu, Dawei Li, Yuchen Liu, Bin Sheng, Andrzej Grzybowski, Tien Yin Wong

**Affiliations:** 1 School of Clinical Medicine, Tsinghua Medicine, Tsinghua University, Beijing, 100084, China.; 2 Beijing Visual Science and Translational Eye Research Institute (BERI), Beijing Tsinghua Changgung Hospital, Tsinghua Medicine, Tsinghua University, Beijing, 100084, China.; 3 Eye Center, Beijing Tsinghua Changgung Hospital, Beijing, 102218, China.; 4 Center for Eye Research Australia, Royal Victorian Eye and Ear Hospital, Melbourne, VIC, Australia; Department of Surgery (Ophthalmology), The University of Melbourne, Melbourne, VIC, Australia.; 5 College of Future Technology, Peking University, Beijing, China.; 6 Key Laboratory for Biomechanics and Mechanobiology of Ministry of Education, Beijing Advanced Innovation Center for Biomedical Engineering, School of Biological Science and Medical Engineering, Beihang University, Beijing, China.; 7 Shanghai International Joint Laboratory for Intelligent Prevention and Treatment of Metabolic Disorders, Department of Computer Science and Engineering, School of Electronic, Information, and Electrical Engineering, Shanghai Jiao Tong University, Department of Endocrinology and Metabolism, Shanghai Sixth People's Hospital Affiliated to Shanghai Jiao Tong University School of Medicine, Shanghai Diabetes Institute, Shanghai Clinical Center for Diabetes, Shanghai, China.; 8 MOE Key Laboratory of AI, School of Electronic, Information, and Electrical Engineering, Shanghai Jiao Tong University, Shanghai, China.; 9 Department of Ophthalmology, University of Warmia and Mazury, Olsztyn, Poland.; 10 Institute for Research in Ophthalmology, Foundation for Ophthalmology Development, Poznan, Poland.; 11 Singapore Eye Research Institute, Singapore National Eye Center, Singapore.

**Keywords:** artificial intelligence, deep learning, retinal imaging, systemic prediction, color fundus photos.

## Abstract

Retinal images provide a non-invasive and accessible means to directly visualize human blood vessels and nerve fibers. Growing studies have investigated the intricate microvascular and neural circuitry within the retina, its interactions with other systemic vascular and nervous systems, and the link between retinal biomarkers and various systemic diseases. Using the eye to study systemic health, based on these connections, has been given a term as oculomics. Advancements in artificial intelligence (AI) technologies, particularly deep learning, have further increased the potential impact of this study. Leveraging these technologies, retinal analysis has demonstrated potentials in detecting numerous diseases, including cardiovascular diseases, central nervous system diseases, chronic kidney diseases, metabolic diseases, endocrine disorders, and hepatobiliary diseases. AI-based retinal imaging, which incorporates established modalities such as digital color fundus photographs, optical coherence tomography (OCT) and OCT angiography, as well as emerging technologies like ultra-wide field imaging, shows great promises in predicting systemic diseases. This provides a valuable opportunity for systemic diseases screening, early detection, prediction, risk stratification, and personalized prognostication. As the AI and big data research field grows, with the mission of transforming healthcare, they also face numerous challenges and limitations both in data and technology. The application of natural language processing framework, large language model, and other generative AI techniques presents both opportunities and concerns that require careful consideration.

In this review, we not only summarize key studies on AI-enhanced retinal imaging for predicting systemic diseases but also underscore the significance of these advancements in transforming healthcare. By highlighting the remarkable progress made thus far, we provide a comprehensive overview of state-of-the-art techniques and explore the opportunities and challenges in this rapidly evolving field. This review aims to serve as a valuable resource for researchers and clinicians, guiding future studies and fostering the integration of AI in clinical practice.

## Introduction

The retina offers a unique, noninvasive, in-vivo visualization of the human body's vasculature and neural tissues 1, serving as a window to the general health. The retinal nerve fibers are essentially an extension of the central nervous system (CNS) axons, and the retinal ganglion cells (RGCs) display typical properties of CNS neurons 2. The retinal blood vessels, densely located in the fundus, not only mirror the features and regulatory mechanism of blood vessels throughout the body, but also act as indicators of general health. Given its vascularization and metabolic activities, the retina provides important clues on the presence of systemic diseases 3.

Over more than 100 years ago, Marcus Gunn described retinal vascular signs in patients with hypertension, kidney disease and stroke, marking the beginning of retinal examination as a source of important clues to systemic health 4. At that time, Polish ophthalmologist Xavier Galezowski (1832-1907), one of the pioneers in the use of fundus examinations for the diagnosis of central nervous system disorders, published one of early textbooks on this subject and coined a term of cerebroscopy for this examination 5, 6. In the last decades of 20^th^ century, studies provided evidence of RGCs degeneration and retinal nerve fiber layer (RNFL) damages in Alzheimer's disease (AD) (neurodegenerative diseases) 7-11, and pathological findings in the retinal vasculature of stroke (cerebral hemorrhage and infarction) 10. This evidence indicates that retinal neural and vascular changes likely exist in the early stage even during asymptomatic period, when they are not distinguishable or cannot be detected by conventional diagnostic methods. These changes can be detected and used as biomarkers for systemic disorders. Insights from many current studies have also proven the correlation between retinal images and the risks of dementia and stroke, which are associated with neural and vascular abnormality, respectively 12-14. These studies laid the foundation for using retinal images to predict systemic diseases.

On the other hand, the rapid updates of technology brought the chance to boost the development of this area, making the previous evidences been taken into clinical practice. In the past decades, advances in information technology have made artificial intelligence (AI) come in quantum leaps in nearly every aspect of our lives, including the medical field. AI, as computer systems capable of performing complex tasks that historically only human could do 15. Machine learning (ML), a subfield of AI uses algorithms trained on datasets to create self-learning models that can predict outcomes and classify information without human intervention 16. ML has been widely used in medical AI. Deep learning (DL), a branch of ML, trains computers to process information in a way that mimics human neural processes and is composed of a neural network with three or more layers (input layer, hidden layers, output layer) 17, 18. Recently, deep convolutional neural networks (CNN), a specialized type of DL technique optimized for images, have produced highly accurate algorithms capable of diagnosing diseases from medical images with accuracy comparable to human experts 19. Leveraging these remarkable advancements, subtle extraction and analysis of retinal images have demonstrated good efficacy in detecting various diseases.

In 2012, Lambin *et al.* first proposed the concept of “radiomics”, the high-throughput extraction of image features from radiographic images. The combination of medical imaging and smart automated or semi-automated software offers the potential to revolutionize quantitative imaging. This approach has paved the way for the integration of AI and medical images in medical diagnosis, treatment and prediction, holding great promises for the future 20, 21.

Ophthalmology is now a field that relies heavily on advanced imaging techniques. Digital color fundus photograph (CFP) is the most common examination, providing detailed images of the retina, optic nerve head, and blood vessels. Optical coherence tomography (OCT) offers cross-sectional details of the retina and choroid, imaging structures at different depths within tissues. OCT angiography (OCTA), a relatively new technology, can show vasculature and structure in specific single layers, imaging and quantifying blood vessels at different depths within tissues. OCT images can serve as biomarkers for neuronal pathways, due to their detailed imaging of RGCs, the RNFL, the inner plexiform layer (IPL) and other neural layers structures 11, while CFP and OCTA can be accessed for vascular pathways 22. Besides, emerging technologies such as ultra-wide field (UWF) imaging provide a comprehensive view of the fundus, capturing details beyond the macula area. As retinal visualization techniques continue to evolve, they will increasingly be applied in retinal biomarkers extraction using AI techniques.

Using the eye to study systemic health, based on these connections, has been given a term as “oculomics”. The term “oculomics” was first proposed in 2020 by Wagner *et al.* 23, indicating a comprehensive understanding of the macroscopic, microscopic, and molecular features associated with health and disease within the eye. With further insights and advancements, oculomics has expanded beyond mere observation, to integrate big data and AI.

Numerous studies have explored the applications of AI-enhanced retinal imaging in predicting different systemic diseases. Even though some of the studies tried to explain how AI works in this period, with interpretative heat map, we still have uncharted parts in this work. It is crucial to know how AI works in oculomics, and we need more investigation on the studies to explore more on the potential explanations.

Therefore, based and progressed on the methods of published reviews in this study area 3, 24-29, we conducted an extensive literature search using the databases “PubMed”, “Medline”, and “Embase” up to May 2024. Search terms include retinal/fundus imaging/image/photo/photograph, OCT, OCTA, UWF; AI, ML, DL, CNN; oculomics, systemic diseases/disorders, and various systemic diseases names. We have extracted the key information of key studies (See Table S1 in the electronic supplementary material for details). We provide a classified summary of key studies on AI-enhanced retinal imaging in predicting systemic diseases, with discussing the mechanisms underlying oculomics and highlighting the challenges and opportunities in this area.

### Cardiovascular Diseases (CVD)

Recent studies have explored the potential of retinal imaging in predicting CVD risks with promising results, marking a significant advancement in medical diagnostics. Rim *et al.* 30 employed CFPs to assess coronary artery calcium (CAC) scores, demonstrating a notable correlation with the risk of fatal cardiovascular events. This approach not only mirrors the predictive capability of traditional computerized tomography (CT)-measured CAC but also enhance risk prediction accuracy when integrated with established cardiovascular risk models, particularly for patients at borderline or intermediate risk levels. Cheung *et al.* 31 developed a fully automated AI/DL-based retinal vessel software (SIVA-DLS), comparing its performance with SIVA-human (retinal-vessel calibre measured by expert human graders) to predict CVD risks. They found narrower arteriolar caliber was associated with higher blood pressure, while wider venular caliber was linked to higher body mass index (BMI), hemoglobin (Hb) A1c, and smoking. SIVA-DLS demonstrated similar associations with CVD risk factors as human measurements and other software's performance. Specifically, a narrower arteriolar diameter and a wider venular diameter were associated with a higher risk of incident CVD. Poplin *et al.* 19 predicted major adverse cardiovascular events (MACE) within 5 years using CFPs, achieving an area under the receiver operating characteristic (AUROC) of 0.73, and compared this model with the SCORE risk calculator. Additional research has applied retinal imaging to further understand the connection to CVD risk factors, traditional cardiovascular examination results or diagnosis standard, diseases detection and future incident risks.

### Cerebrovascular Diseases (CeVD)

The CFPs combined with superficial and deep enface OCTA images 32 or CFP-originated vasculometry 33, 34 have been used to generate the AI algorithms for stroke events prediction. Different wavelength fundus photos have also been applied and compared in predictive work 34. In addition to stroke prediction, Hong *et al.* 35 were the first to predict and stage moyamoya disease using a CFP-based algorithm. Notably, the retinal age gap, which was defined as the difference between predicted biological age based on CFP and chronological age, has been used to predict the hazard ratio (HR) for stroke events 36. It has been shown that the highest stratified population by retinal age gap has an HR of 2.37 compared to the lowest. Another significant predictor is white matter lesions (WMLs) from magnetic resonance imaging (MRI) which reflects the severity of CeVD, used as the output prediction of CFP-based algorithm 37. Retinal imaging can thus be used to output CeVD brain imaging results, directly detect CeVD, or predict future stroke incidents.

### Neurodegenerative Diseases

Cheung *et al.* 38 used AI/DL algorithm to detect AD-dementia of 11 multi-ethnic, multi-country studies with 12949 retinal photos (648 AD-dementia cases vs 3240 controls). The algorithm used CFPs to detect AD with AUROC 0.93, whereas detect amyloid β-positive AD with AUROC 0.73-0.85. Another study 39 developed a model by CFPs and clinical dementia rating global scores. The model can automate estimate central retinal arteriolar equivalent (CRAE) and central retinal venular equivalent (CRVE), give the association with cognitive and incident dementia, which may be useful as a risk stratification tool. The study also showed narrower arteriolar diameter and wider venular diameter associated with higher risk of incident dementia. That proves the possibility AI/DL retinal vessel software predicts dementia. OCT images have been used to predict AD, Parkinson's disease (PD), multiple sclerosis (MS) 40-42 and long-term prediction of MS disability course 43. OCTA images combined with OCT in predicting mild cognitive impairment (MCI) showed model efficacy with area under the curve (AUC)=0.693-0.960 44. Other studies combined CFPs and metadata or risk factors to generate the models with better task performance (Table S1). Various retinal imaging and the multi-modality imaging have been widely used in different neurological diseases based on the intense connection between eye and brain.

### Schizophrenia and Other Psychiatric Diseases

Appaji *et al.* 45 developed a model to predict schizophrenia using CFPs. Their model was trained to distinguish individuals with schizophrenia from healthy subjects, achieving an AUC of 0.98. Although some literature mentioned that psychiatric diseases, such as bipolar disorder, have exhibit vascular abnormalities that can be detected in retinal images 46, further research and AI-based algorithms are still needed. Recent studies have proposed that changes detected by ML-based electroretinography (ERG) could serve as sensitive indicators of autism spectrum disorder 47. It is hoped that more psychiatric diseases, including depression and anxiety that have seen a rise in prevalence in recent years, will be explored in AI-retinal imaging studies.

### Renal Diseases

Kidney failure is typically defined by estimated glomerular filtration rate (eGFR) levels, several models have been developed to predict chronic kidney disease (CKD), diabetic kidney disease (DKD) or diabetic nephropathy (DN). Sabanayagam *et al.* 48 used CFPs and a combined model trained with risk factors to predict CKD, achieving AUCs of 0.911 and 0.938. Kang *et al.* 49 predicted early renal function impairment with an AUC of 0.81. Similar studies have been conducted to directly predict eGFR, early CKD, CKD, and DKD labels 50-54. Liu *et al.* 55 used OCT images to predict DN, with an accuracy of 91.68%, a sensitivity of 89.99% and specificity of 92.18%. Zhang *et al.* 56 also studies retinal age, calculating the HR of incident kidney failure using cohort data. Similarly, Joo *et al.* 57, used the Reti-CKD score, derived from CFPs in a cohort, to calculate the HR of CKD incidence. When the population was stratified by the Reti-CKD score, the HR were 3.68 and 9.36 in 2 cohorts from the highest to the lowest scores. The retinal age gap and Reti-CKD score can successfully be applied as predictors of renal diseases incidence, showing great promise and inspiring more predictors contribution.

### Metabolic Diseases

Algorithms based on CFPs have shown good performance in predicting hypertension, hyperglycemia, dyslipidemia with AUCs of 0.766, 0.880 and 0.703 respectively 58. Benson *et al.*, have demonstrated that diabetes and diabetic peripheral neuropathy (DPN) can be detected using AI-CFPs models 51, 59-61. Xiang *et al.* 62 innovatively combined CFPs with traditional Chinese medicine characteristics (tongue and pulse conditions) to predict diabetes, which inspired more hybrid models related to metabolic factors to enhance the performance of standalone AI models on retinal imaging. Studies on retinal age gap studies have also revealed a relationship between retinal age gap and metabolic syndrome and inflammation. The stratified population shows different odds ratios (ORs) indicating that retinal age gap could become a promising variable 63.

### Hepatobiliary Diseases

Xiao *et al.* 64 used CFPs to generate a screening model for hepatobiliary diseases and an identification model for 6 specific hepatobiliary diseases (including liver cancer, liver cirrhosis, chronic viral hepatitis, non-alcoholic fatty liver disease, cholelithiasis, hepatic cyst). The AUROCs for screening and identification were 0.68, 0.84, 0.83, 0.62, 0.70, 0.68 and 0.69 respectively. Xiao's model provided a non-invasive, convenient, and complementary method. In the same study, they also used slit-lamp photos to train models, which achieved good performance as well.

### Anaemia

Anaemia is typically diagnosed by measuring Hb concentration through a blood assay test, which offered a quantitative variable as the ground truth. Some studies use anaemia diseases labels diagnosed by doctors. Haematocrit and red blood cell count have also been used as auxiliary variables in predicting anaemia. Mitani *et al.* 65 used CFPs, metadata (race or ethnicity, age, sex and blood pressure) and combined data to predict Hb concentration and diagnose anemia. The combined model showed better performance with Hb prediction (mean absolute error [MAE]=0.63g/dl), and achieved an AUC of 0.88 for detecting anemia and 0.95 for moderate anaemia. Rim *et al.* 1 also used CFPs to predict Hb, with an MAE of 0.79g/dl. UWF images have been used by Zhao *et al.* 66 to predict Hb and anaemia, achieving an MAE of 0.83g/dl and an AUC of 0.93 for anaemia. OCT images from anaemia patients and normal subjects were used by Chen *et al.* 67 and Wei *et al.* 68 to train classifiers, achieving accuracies of 0.8358 and 0.9865 respectively. Automated non-invasive anaemia screening based on retinal imaging represents a breakthrough, especially for individuals with conditions such as diabetes, where anaemia can increase morbidity and mortality risks 65, 69.

### How AI Work with Retinal Imaging and Systemic Diseases Predicting?

#### Three Pathways Linking Retina and Body

AI-based retinal imaging has been intensively explored in predicting disorders across different systems or organs. To sum up, there are three pathways linking retina and body. First, when focusing on the onset of diseases such as etiology, pathogenesis and inducing factors, traditional risk factors like blood pressure, age, BMI, smoking, etc. can be used as predictors. Additionally, there are less understood or unidentified risk factors including genetic and inflammation factors. We can directly estimate the diseases risk factors, thereby linking these factors with different diseases. Second, we can predict established examination results that can be used to monitor or diagnose diseases, such as carotid ultrasound, cardiac computerized tomography, and MRI results. Retinal imaging offers a straightforward, non-invasive, and cost-effective alternative for replacing the biomarkers typically identified by these conventional methods. Third, we can directly estimate specific clinical diseases, distinguish between diseased and non-diseased individuals. Most notably, when adding metadata or risk factors into retinal imaging to develop hybrid algorithms, the predictive model showed improved effectiveness in predicting tasks.

#### New Parameters as a Bridge between Retina and Body

When we use AI-based retinal imaging to estimate risk factors or traditional biomarkers, we can also generate new variables derived from the predictive results. New variables originated from retinal images, such as Reti-Age, Reti-CKD, Reti-CAC, can be analyzed for their relationships with diseases outcomes. Another part of generating parameters is the direct retinal parameters, including caliber, geometry, fractals, bifurcation, tortuosity, and artery-vein nicking. While these parameters can be observed by ophthalmologists, they are better accessed and quantified by AI system for greater accuracy and subtlety, making these parameters quantitative and comparable. These direct and indirect variables extracted from retinal images using AI can be used to find correlations with clinical diseases through mathematical and statistical ways, rather than being limited to descriptive and qualitative analysis.

#### Current Retinal Imaging Predicting Future

With the availability of longitudinal data, it is possible to track the incidence of disease, mortality, and severe outcomes over time. By incorporating longitudinal data into AI model, we can predict the incidence and progression of diseases from baseline images. This enables risk stratification based on certain variables, which is particularly inspiring.

**Figure 1:**
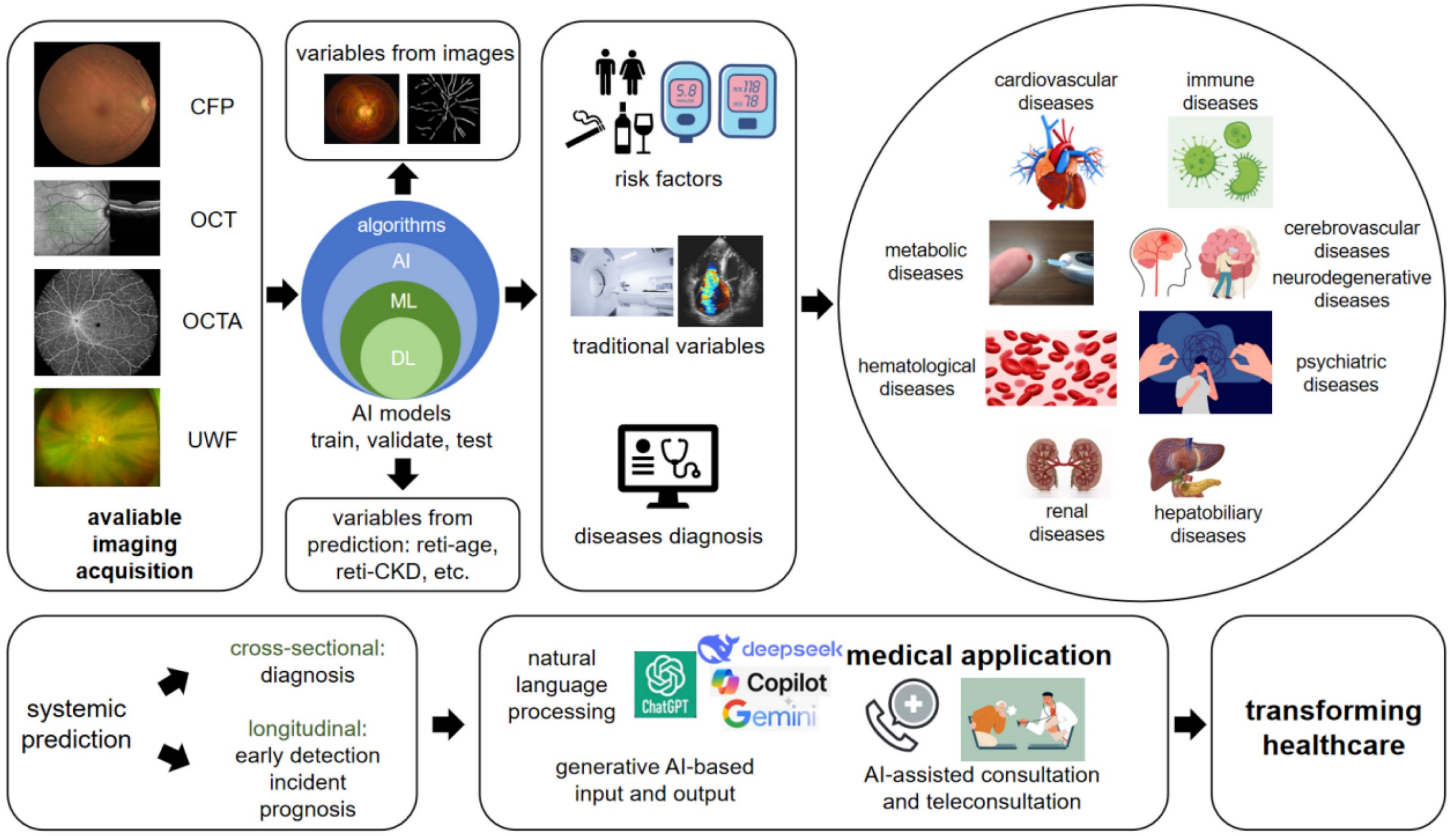
** The overview of how AI-enhanced retinal imaging predicting systemic diseases and the future considerations.** Based on various modalities of available retinal imaging, through the pre-trained AI algorithms, we can predict risk factors, traditional variables and diseases diagnosis, which already applied in disorders from nearly all systems, including cardiovascular, metabolic, hematological, renal, hepatobiliary, psychiatric, cerebrovascular, neurodegenerative and immune diseases. The prediction includes not only diagnosis, but also early detection, incident and prognosis of the disorders based on the training on longitudinal data. The emerging new era of generative AI brings promising opportunities on medical application and healthcare transforming times. CFP: color fundus photo; OCT: optical coherence tomography; OCTA: optical coherence tomography angiography; UWF: ultra-wide field; AI: artificial intelligence; ML: machine learning; DL: deep learning.

Figure 1 shows the ideas and workflow of current AI-based retinal imaging for systemic diseases prediction. Here we summarized how AI works in this study area, also provided thoughts on how to begin an AI-oculomics study.

### Future Outlook and Considerations on AI-oculomics Studies

#### Challenges in Data

Despite remarkable advancements in this area, there are still many challenges and next steps to be determined. Firstly, the accuracy of AI predictions heavily relies on the quality and quantity of the available data. Limited or biased data may result in less accurate predictions. Additionally, AI models may struggle with rare or complex diseases that are underrepresented in the training sets. We need more real-world prospective studies. Another issue is, longitudinal data is much more limited compared to cross-sectional data. Large datasets containing ophthalmological imaging such as those from the UK biobank, Qatar biobank are still scarce. Local hospitals and health centers should be encouraged to enhance long-term and multi-modality data collection. This effort could significantly contribute to the global early detection and incidence and prognosis of systemic diseases. Khan *et al.* 70 reviewed the publicly available datasets for ophthalmological imaging, highlighting the need for more comprehensive datasets.

Currently, systemic biomarkers studied through CFPs are more common than those obtaied via OCT, OCTA and other novel modalities. There is a need for more algorithms to explore the feasibilities of these advanced imaging modalities. In addition, OCT can provide data on choroidal thickness, a crucial parameter reflecting choroidal circulation. However, this area is still an underexplored and requires more advanced imaging technologies like sweep source OCT (SS-OCT) for further investigation. We need to pay more attention to high-resolution instruments and reliable data.

The current study is mostly based on single image. It is still difficult to combine different modalities imaging into one model. In comparison with physicians' work, which typically use multi-modality imaging to achieve a comprehensive understanding of the disease, the AI's capability in this regard remains incomplete (Table 1).

#### Challenges in Technology

Although AI can assist in detecting potential diseases or anomalies, it is crucial to involve human experts in reviewing and interpreting the results to ensure accurate diagnosis and appropriate treatment decisions. As we consider the future use of the AI technology, it is important to recognize that it will be used by a wide range of professionals, including ophthalmologists, optometrists, general physicians, neurologists, cardiologists, or patients themselves. The aim of this digital technology is to reduce the need for expensive complex examinations, providing an early detection method for diseases that is non-invasive, easy and cost-effective. However, the technology is still in the development stage, and more robust modeling is needed. The challenges of modeling also embody on integration of immense clinical and high throughput biological data, multi-task learning on disease diagnoses, therapy and outcome predictions and cost-effective computing capabilities. Hence, increasing the data by technological means, developing advanced multi-modal interpretative networks for prediction of multi-source ocular histology images, increasing the number of AI models for continuous learning and self-optimization, become much needed.

### Future Directions with Generative AI and Foundation Models

Based on the previous findings in research, and numerous challenges and opportunities in data and technology. We propose that the novel technology in AI, including generative AI techniques and foundation models, can make up for the weaknesses in research and applications. These could give us inspirations in future directions and solve the existing problems. How these technologies apply into real medical situations and transform the healthcare? We explained in the following part and expressed our thoughts on how they could work in ophthalmology.

Recent years have witnessed significant advancements in the state of art AI technology. Generative AI includes a wide array of applications, including the generation of images, videos, text, sound, software codes, virtual environments, designs and even drug compound 71. It is based on various techniques, including DL, generative adversarial networks (GAN), autoencoders, variational autoencoders and etc. These models can be utilized in retinal images research and provide robust support to address the limitations of current research methodologies. You *et al.* 72 conducted a survey on studies using GAN in ophthalmology image domain and introduced its performance on image synthesis and image-to-image translation. It helps extend datasets and modalities in research, particularly in scenarios with limited sample size and varying images quality and opens up new research possibilities through translational work.

Conversational large language model is a kind of generative AI, that makes sense of natural language. Large language model (LLM), including DeepSeek, GPT-o1 and 4o (OpenAI), LLaMA (Meta), PaLM (Google), BERT (Google), Gemini (Google), Copilot (Microsoft) and Claude (Anthropic) have recently drawn considerable interest from journalists, policymakers, and scholars across fields 73, 74. By learning the probabilities of words sequences from extensive corpus of text, these models with billions of parameters, can respond to free-text queries without needing task-specific training 75.

LLMs have shown immense promise in the medicine field and are rapidly being integrated into clinical practice. In clinical situations, it can be applied to automatically communicate with patients, draft clinical notes, and even provide clinical suggestions. Due to their ability to handle free-text input, LLM-based tools can manage documents, recognize and extract information from text databases in clinical studies. Therefore, it is useful to build the relevant medical LLMs. The implements are not only in the human-machine interface, automatically interactions with data collection part and model/program users, but also in model/program developing procedures, being utilized to filter and administrate text parameters or labels and helping enhance and accelerate the multi-modality model inputting work.

Hence, we propose the clinical translation of generative AI-based input and output. 1) AI-assisted consultation: Nature language processing (NLP) framework combined with DL models, clinically relevant information and features can be extracted. Then, different AI-systemic prediction models can provide diagnostic systems for various systemic diseases and output medical recommendations. 2) AI-assisted teleconsultation. Currently, with AI-assisted consultation been used in different websites and APPs by chatbots, we have seen that there are already merchant products catering to the market. It is hoped that more generative AI approaches will make subjects more attractive and real experience. Recent SORA is also a breakthrough in video generation, which can be applied into human-computer interaction procedures (Figure 1).

**Figure 2:**
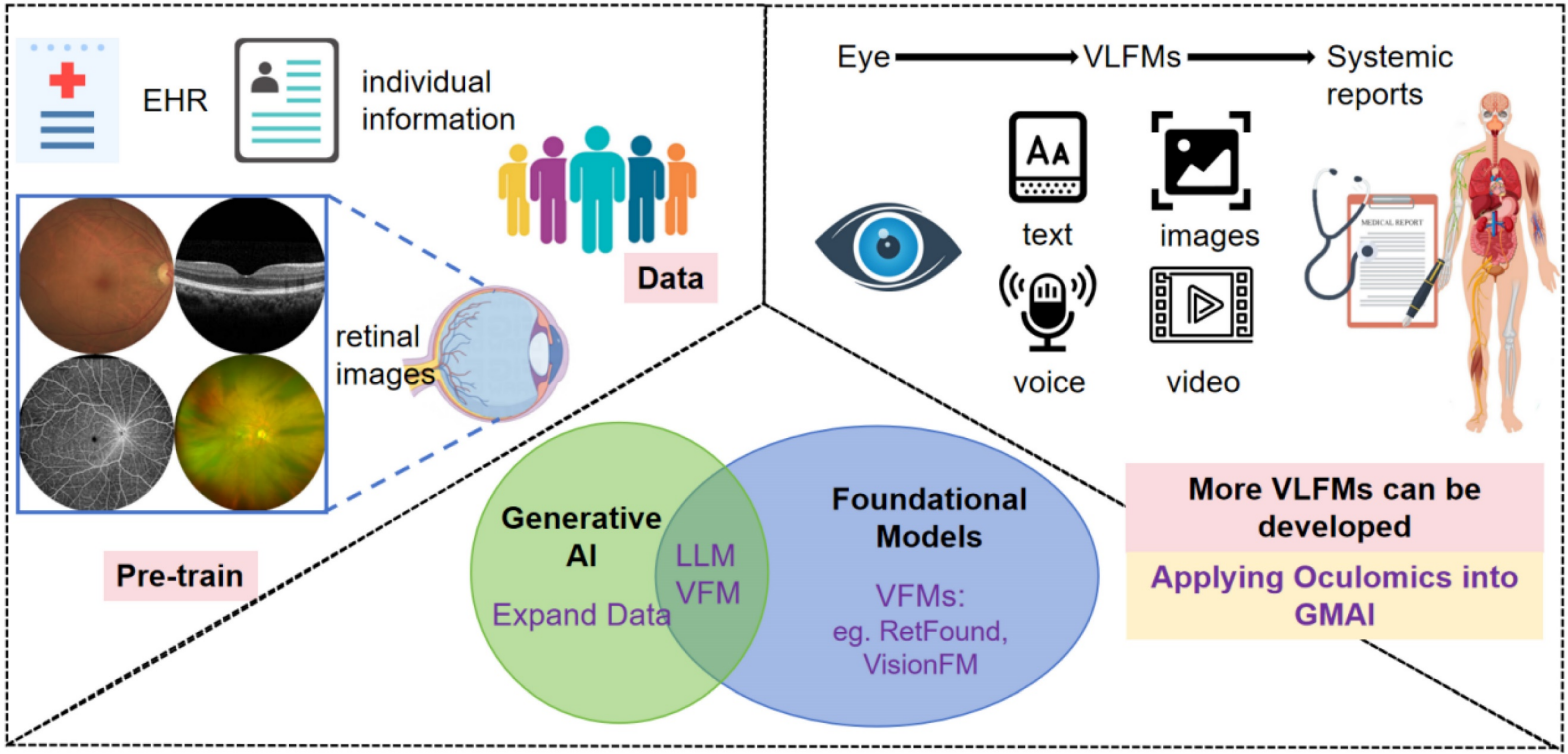
** Systemic disorders diagnosed on eye images developed AI models.** Previous AI algorithms trained on EHR, individual information, and retinal images of certain population, that sets good basics to the coming new AI models. With the development of generative AI, multimodality new VLFMs may contain text, voice, images and video, linking previous AI models into a friendly human-computer interaction mode, applying oculomics studies into the GMAI system, potentially transforming ophthalmology healthcare. EHR: electronic health record; AI: artificial intelligence; LLM: large language model; VFM: vision foundational models; VLFM: vision-language foundational models; GMAI: generalist medical artificial intelligence.

RETFound, one of the first AI foundation models in healthcare, and the first in ophthalmology, was developed using millions of eye scans from the National Health Service (NHS) 76. Zhou *et al.* 77 provided a generalizable solution that enables broad clinical AI applications from retinal imaging, predicting conditions such as ischaemic stroke, myocardial infarction, heart failure, PD, in addition to ophthalmic diseases. This foundation model has inspired more foundation models, AI-agents even artificial general intelligence developing in this area, especially vision-language foundational models (VLFM) that combine LLM, progressing the prediction work wider-use, more user friendly and competitive of usability 78. The developed AI models will apply oculomics studies into the generalist medical artificial intelligence (GMAI) system 79, potentially transforming ophthalmology healthcare, which is much inspiring (Figure 2).

In addition, when we face formal clinical trials in following steps of AI models application in clinic, more ethical questions should be taken into consideration. Currently, no generative AI systems have been reviewed by the United States Food and Drug Administration (FDA) 71. The problems contain data breach, privacy risks, medical responsibility, and supervision. Meanwhile, technically, it is not a totally correct system and still retains bias to some extent. The risks of misdiagnosis may bring more controversial problems (Table 1).

### AI-oculomics Applications in Real World

We are thrilled to see emerging AI-related start-ups focusing on realizing the applications. It has been truly applying AI-oculomics studies' findings into practice. Singapore Eye Lesion Analyser (SELENA+) is a deep-learning AI software system that can detect potential threatening eye conditions accurately and efficiently 80. It initially focused on diabetic retinopathy (DR) screening, then expanded to encompass the screening of multiple chronic diseases, especially in CVD. AIFUNDUS is another software that can detect DR and other retinal diseases. Combining with their health risk assessment system, it can provide health report related to CVD, metabolic diseases and nervous system diseases 81. The EyeArt system 82 and LumineticsCore™ 83 are also AI diagnostic systems, maily focusing on DR.

There are still bunches of obstacles to face when the new start-ups make these technologies as products in our real life. Current bussiness modes are mostly business to business (B2B). The clients are mostly hospitals, pharmacy chains, optical chains, health screening outlets, and primary community healthcare settings. Apart from the lack of AI industrial standards and difficulty in registration and ethic review for AI products, whether doctors and patients can actually use the results report, and who will pay for the screening and detection under various coutries' healcare policies, are still difficulties that start-ups need to find ways to survive. Expanding the bussiness mode into the business to consumer (B2C) market with the launch of a dedicated app, offering personalized health insights and enhance customer engagement, still need a long way to explore.

However, as the global prevalence of chronic illnesses continues to rise, healthcare systems worldwide are increasingly challenged by escalating costs. Acting as an all-in-one (retina imaging) solution for the detection of multiple chronic diseases, we believe the existing and futural updating products will significantly streamline the screening process, eliminating the need for multiple tests. This not only boosts productivity and reduces costs but also expedites the delivery of critical results to patients.

## Conclusion

Overall, while there are limitations, AI is continuously evolving and holds great potential in transforming healthcare. AI has the potential to accelerate existing forms of medical analysis, but its algorithms require further testing to be fully trusted. For now, the idea of an AI doctor independently making new diagnoses without human oversight remains a distant prospect - like decades away rather than years. The path toward a new AI-powered paradigm is needed.

With the emerging concept of “oculomics” and “retinomics” 84, more and more research are leveraging AI-derived methods to explore correlations between systemic biological factors and retina. The role of AI in retinal imaging for predicting systemic diseases is thus becoming more promising and sustainable.

## Figures and Tables

**Table 1:** ** The current discussion and considerations on AI-oculomics studies.** It is a double-edged sword, that the opportunities are in challenges, while challenges also imply the opportunities. We summarized the key points, giving a quick highlight of the discussion on the study.

	Challenges	Opportunities
**Data**	Limited data, limited real-world studies in rare diseases, limited longitudinal cohort and comprehensive biobanks.	Call for more real-word studies in local hospitals and health centers. Physicians need the realization of biobank and data integration. Call for more consortium actions to combine data.
	Limited modalities and we need more advanced imaging equipment involved.	Advanced imaging like SS-OCT, OCTA, UWF, Vis-OCT, PS-OCT, PD-OCT, AO-OCT, oximetry and etc. can be candidates in futural studies. Other functional examination also can be candidates in AI-based studies, such as microperimetry, visual acuity, intraocular pressure.
	Limited multi-modality algorithms.	The physicians' work are based on the comprehensive understanding of whole body. AI still has the potential to help with the process based on multi-modality work.
**Technology**	Controversial results accuracy, interpretability, usability and reliability.	Call for more studies to strengthen oculomics concept through evidence, validation, mechanistic understanding and human-machine interaction testing.
	No generative AI systems have been reviewed by FDA.	Call for more RCT clinical trials over the world.

AI: artificial intelligence; SS-OCT: sweep source optical coherence tomography; OCTA: optical coherence tomography angiography; UWF: ultra-wide field; Vis-OCT: visible wavelength OCT; PS-OCT: polarization-sensitive OCT; PD-OCT: polarization-diversity OCT; AO-OCT: adaptive optics OCT; FDA: United States Food and Drug Administration; RCT: randomized controlled trial.

## Abbreviations

AD: Alzheimer's disease
AI: artificial intelligence
AUC: area under the curve
AUROC: area under the receiver operating characteristic
B2B: business to business
B2C: business to consumer
BMI: body mass index
CAC: coronary artery calcium
CeVD: cerebrovascular diseases
CFP: color fundus photograph
CKD: chronic kidney disease
CNN: convolutional neural networks
CNS: central nervous system
CRAE: central retinal arteriolar equivalent
CRVE: central retinal venular equivalent
CT: computerized tomography
CVD: cardiovascular diseases
DKD: diabetic kidney disease
DL: deep learning
DN: diabetic nephropathy
DPN: diabetic peripheral neuropathy
DR: diabetic retinopathy
eGFR: estimated glomerular filtration rate
ERG: electroretinography
FDA: Food and Drug Administration
GAN: generative adversarial networks
GMAI: generalist medical artificial intelligence
Hb: hemoglobin
HR: hazard ratio
IPL: inner plexiform layer
LLM: large language model
MACE: major adverse cardiovascular events
MAE: mean absolute error
MCI: mild cognitive impairment
ML: machine learning
MRI: magnetic resonance imaging
MS: multiple sclerosis
NHS: National Health Service
NLP: nature language processing
OCT: optical coherence tomography
OCTA: OCT angiography
ORs: odds ratios
PD: Parkinson's disease
RGCs: retinal ganglion cells
RNFL: retinal nerve fiber layer
SELENA+: Singapore Eye Lesion Analyser
SIVA-DLS: Singapore "I" vessel assessment-deep learning system
SS-OCT: sweep source OCT
UWF: ultra-wide field
VLFM: vision-language foundational models
WMLs: white matter lesions
