# Supplementary material for: Artificial intelligence-enhanced retinal imaging as a biomarker for systemic diseases
Source: Theranostics. 2025 Feb 18;15(8):3223–33. doi: 10.7150/thno.100786 (PMC11905132; doi:10.7150/thno.100786)
Supplement: Supplementary file 1 — Supplementary table. [file thnov15p3223s1.pdf]

Supplementary Material

Artificial intelligence-enhanced retinal imaging as a biomarker for systemic diseases

Jinyuan Wang[1,2,3], Ya Xing Wang[2], Dian Zeng[1], Zhuoting Zhu[4], Dawei Li[5], Yuchen Liu[6], Bin Sheng[7,8], Andrzej Grzybowski[9,10], Tien Yin Wong[1,2,11]

[1] School of Clinical Medicine, Tsinghua Medicine, Tsinghua University, Beijing, 100084, China.
[2] Beijing Visual Science and Translational Eye Research Institute (BERI), Beijing Tsinghua Changgung Hospital, Tsinghua Medicine, Tsinghua University, Beijing, 100084, China.
[3] Eye Center, Beijing Tsinghua Changgung Hospital, Beijing, 102218, China.
[4] Center for Eye Research Australia, Royal Victorian Eye and Ear Hospital, Melbourne, VIC, Australia; Department of Surgery (Ophthalmology), The University of Melbourne, Melbourne, VIC, Australia.
[5] College of Future Technology, Peking University, Beijing, China.
[6] Key Laboratory for Biomechanics and Mechanobiology of Ministry of Education, Beijing Advanced Innovation Center for Biomedical Engineering, School of Biological Science and Medical Engineering, Beihang University, Beijing, China.
[7] Shanghai International Joint Laboratory for Intelligent Prevention and Treatment of Metabolic Disorders, Department of Computer Science and Engineering, School of Electronic, Information, and Electrical Engineering, Shanghai Jiao Tong University, Department of Endocrinology and Metabolism, Shanghai Sixth People's Hospital Affiliated to Shanghai Jiao Tong University School of Medicine, Shanghai Diabetes Institute, Shanghai Clinical Center for Diabetes, Shanghai, China.
[8] MOE Key Laboratory of AI, School of Electronic, Information, and Electrical Engineering, Shanghai Jiao Tong University, Shanghai, China.
[9] Department of Ophthalmology, University of Warmia and Mazury, Olsztyn, Poland.
[10] Institute for Research in Ophthalmology, Foundation for Ophthalmology Development, Poznan, Poland.
[11] Singapore Eye Research Institute, Singapore National Eye Center, Singapore.

*Correspondence: Tien Yin Wong, wongtienyin@tsinghua.edu.cn

Supplementary Table 1. The key information of the key studies related to AI-based retinal imaging predicting systemic diseases. We summarized systems, authors and publication time, development datasets, validation datasets, external datasets, input, model, output, metrics, internal results and external results, which provided a thorough reference for the academics.

Systemic Diseases	Author,<br>Publication Time	Country	Development Datasets	Validation Datasets	External Datasets	Input	Model	Output	Metrics	Internal Results	External Results
Cardiovascular Diseases	Nele Gerrits, Jun. 2020[1]	Belgium	Qatar Biobank(12000 images of 3000 subjects)--80%	--20%		CFP	MobileNet-V2	age, sex, SBP, DBP, Haemoglobin A1c, relative fat mass, testosterone	MAE<br>AUC	age:MAE=2.78 years<br>sex:AUC=0.97<br>SBP:MAE=8.96 mmHg<br>DBP:MAE=6.84 mmHg<br>Haemoglobin A1c:MAE=0.61%<br>relative fat mass:MAE=5.68 units<br>testosterone:MAE=3.76 nmol/L	
	Tyler Hyungtaek Rim, May 2021[2]	Singapore	Severance Hospital of South Korea:5590 images of 2536 subjects		1. Philip Medical Centre of South Korea:18920 images of 8707 subjects<br>2. Cardiovascular and Metabolic Disease Etiology Research Center-High Risk (CMERC-HI):1054 images of 527 subjects<br>3. SEED:17102 images of 8551 subjects<br>4. UK Biobank:95358 images of 47679 subjects	CFP		RetiCAC	AUC	AUC=0.742	concordance index=0.71<br>HR of fatal cardiovascular events=1.33
	Carol Y. Cheung, Jun. 2021[3]	Singapore	SEED(5309 subjects)		5636 subjects<br>HR:59191 subjects	CFP+age, gender, ethnicity, fellow calibre, BMI, MABP, glycated-haemoglobin level, total cholesterol level and smoking at baseline	SIVA-DLS	CRAEb<br>CRAEc<br>CRVEb<br>CRVEc	HR for CVD event	HR=1.08-1.12/per SD	
	Hamada R. H. Al-Absi, Jun. 2022[4]	Qatar	Qatar Biobank(500 subjects:250 CVD:250 controls)			CFP+DXA	AlexNet<br>VGGNet-11<br>VGGNet-16<br>ResNet-18<br>ResNet-34<br>DenseNet-121<br>SqueezeNet-0<br>SqueezeNet-1	CVD	accuracy	accuracy=78.3%	
	Alicja Regina Rudnicka, Dec. 2022[5]	UK	UKB(64144:327 circulatory deaths)<br>UKB(45734:393 incident MI)		European Prospective Investigation into Cancer (EPIC)-Norfolk(5862:201 circulatory deaths)<br>EPIC-Norfolk(4062:265 incident MI)	1. CFP-vasculometry +age, smoking, medical history<br>2. Framingham risk scores(FRS) for confirmed MI	QUARTZ	Circulatory mortality<br>Myocardial infarction	Calibration slope<br>C-statistic<br>R^2	Circulatory mortality:<br>men:<br>Calibration slope=1:0.913<br>C-statistic=1:0.749<br>R^2=1:0.369<br>women:<br>Calibration slope=1:0.857<br>C-statistic=1:0.763<br>R^2=1:0.443<br>Myocardial infarction:<br>men:<br>Calibration slope=1:0.836, 2:1.216<br>C-statistic=1:0.675, 2:0.706<br>R2=1:0.178, 2:0.235<br>women:<br>Calibration slope=1:0.803, 2:1.036<br>C-statistic=1:0.709, 2:0.758<br>R2=1:0.226, 2:0.345	Circulatory mortality:<br>men:<br>Calibration slope=1:1.084<br>C-statistic=1:0.774<br>R^2=1:0.392<br>women:<br>Calibration slope=1:0.872<br>C-statistic=1:0.748<br>R^2=1:0.333<br>Myocardial infarction:<br>men:<br>Calibration slope=1:0.905, 2:1.567<br>C-statistic=1:0.641, 2:0.689<br>R2=1:0.150, 2:0.233<br>women:<br>Calibration slope=1:0.786, 2:0.834<br>C-statistic=1:0.650, 2:0.688<br>R2=1:0.162, 2:0.208
	Grace Lui, Feb. 2023[6]	Hong Kong, China	the Prince of Wales Hospital:115 subjects(HIV)--70%	--30%		CFP+traditional cardiovascular risk factors	ResNet50<br>ARIA<br>RF	coronary atherosclerosis<br>obstructive CAD	AUC<br>sensitivity<br>specificity	coronary atherosclerosis:AUC=0.987, sensitivity=93.0%, specificity=93.2%<br>obstructive CAD:AUC=0.991, sensitivity=95.7%, specificity=97.8%	
	Joseph Mellor, Jul. 2023[7]	UK	Scottish Diabetes Research Network dataset (SDRN-NDS)(24012 T1DM:202843 T2DM)--70%	--30%		CFP	ResNet101	CVD<br>eGFR<br>SBP<br>DR	AUC<br>sensitivity<br>specificity<br>PPV<br>F1	T1DM:<br>AUC=0.822<br>sensitivity=0.867<br>specificity=0.557<br>PPV=0.994<br>F1=0.926<br>T2DM:<br>AUC=0.711<br>sensitivity=0.905<br>specificity=0.282<br>PPV=0.978<br>F1=0.940	

	Cynthia Ciwei Lim, Sep. 2023[8]	Singapore	SEED(860 CKD)10 years			CFP+Established risk factors+eGFR	SIVA-DLS	incident CVD mortality	AUC	AUC=0.760	
Cerebrovascular Diseases	Yimin Qu, Aug. 2022[9]	Hong Kong, China	Shenzhen Traditional Chinese Medicine (SZTCM) Hospital and community(711 subjects, 145 ischemic stroke:86 haemorrhagic stroke:480 controls)--70%	--30%		CFP-retinal variables +clinical variables	ResNet50<br>ARIA<br>RF	ischemic stroke<br>haemorrhagic stroke	sensitivity<br>specificity<br>AUC	ischemic stroke:<br>sensitivity=91.0%, specificity=94.8%, AUC=0.929<br>haemorrhagic stroke:<br>sensitivity=93.0%, specificity=97.1%, AUC=0.951	
	Zhuoting Zhu, Nov. 2022[10]	China	UK biobank(19200 images of 11052 subjects)	test:<br>UKB(35917 subjects)<br>pridict:<br>UKB(35304 subjects)		CFP	Xception	retinal age gap	HR of stroke events	4% increase stroke risk/1 year retinal age gap increase, HR=1.04<br>first quintile/fifth quintile:HR=2.37	
	Samiksha Pachade, Dec. 2022[11]	USA	1. NASA project(112 images:16 cases (15ischemic, 1 haemorrhagic):73 controls)<br>2. +OCT-500(500 subjects) and ROSE(229 octa images)			CFP<br>superficial and deep enface OCT-A images	1. feature engineering (ResNeSt)<br>2. self-supervised learning(ResNet-50)<br>KNNs, decision tree, random forest, multi-layer perceptron (MLP), adaBoost, and Gaussian naive Bayes	stroke	AUC	1. AUC=0.87-0.88<br>2. AUC=0.66=0.81	
	Alicja Regina Rudnicka, Dec. 2022[5]	UK	UKB(63839:446cases)		European Prospective Investigation into Cancer (EPIC)-Norfolk(5708:211cases)	1. CFP-vasculometry +age, smoking, medical history<br>2. Framingham risk scores(FRS) for stroke	QUARTZ	incident stroke	Calibration slope<br>C-statistic<br>R^2	men:<br>Calibration slope=1:0.896, 2:0.908<br>C-statistic=1:0.729, 2:0.736<br>R^2=1:0.315, 2:0.295<br>women:<br>Calibration slope=1:0.860, 2:0.919<br>C-statistic=1:0.753, 2:0.736<br>R^2=1:0.352, 2:0.310	men:<br>Calibration slope=1:0.808, 2:0.819<br>C-statistic=1:0.691, 2:0.682<br>R^2=1:0.213, 2:0.199<br>women:<br>Calibration slope=1:0.780, 2:0.943<br>C-statistic=1:0.714, 2:0.732<br>R^2=1:0.274, 2:0.309
	Liming Shu, Jul. 2023[12]	China	First Affiliated Hospital of Sun Yat-sen University(259 patients)			1. left retinal images<br>2. right retinal images<br>3. the clinical-laboratory signature<br>4. 3+demographic data<br>5. 1+2+4	ResNet-18	the probability of moderate/severe white matter lesions (WMLs)	AUC<br>accuracy<br>precision<br>recall<br>F1 score<br>sensitivity<br>specificity<br>R^2<br>recall rate	AUC=1:0.73, 2:0.94, 3:0.73, 4:0.78, 5:0.95	
	Hui Li, Aug. 2023[13]	China	Beijing Yanhua Hospital and Beijing Xuanwu Hospital of Capital Medical University:<br>150 atrial fibrillation without IS in 1 year and 100 atrial fibrillation with ischemic stroke 1 year			fundus photos<br>1. 548nm wavelengths<br>2. 605nm wavelengths<br>3. 810nm wavelengths<br>4. 1+2<br>5. 1+2+3	Inception V3<br>ResNet50<br>SE50	ischemic stroke	AUC<br>accuracy<br>sensitivity<br>specificity<br>positive predictive value(PPV)<br>negative predictive value(NPV)<br>F1 score	Inception V3:<br>AUC=1:0.913, 2:0.910, 3:0.904, 4:0.945, 5:0.954<br>ResNet50:<br>AUC=1:0.883, 2:0.887, 3:0.863, 4:0.892, 5:0.940<br>SE50:<br>AUC=1:0.878, 2:0.900, 3:0.875, 4:0.920, 5:0.953	
	Lin An, Sep. 2023[14]	China	Guangdong Provincial Hospital of Integrated Traditional Chinese and Western Medicine(409 subjects:133 cases:276 controls))			CFP	ResNet<br>EfficientNet<br>Efficient-attention	CeVD risk	accuracy<br>AUC<br>specificity<br>sensitivity	Efficient-attention:<br>AUC=0.904<br>accuracy=0.834<br>specificity=0.884<br>sensitivity=0.746	
	JaeSeong Hong, Jan. 2024[15]	Republic of Korea	Severance Hospital:322 images of 67 cases:3752 images of 1616 controls--rest	Severance Hospital:<br>test:83 images of 17 cases:83 images of 33 controls		CFP	ResNeXt50	moyamoya disease(MMD) screening<br>MMD staging	AUC<br>accuracy<br>sensitivity<br>F1-score	MMD screening:AUC=94.6%<br>MMD staging:AUC=stage 1:78.9%, stage 2:80.6%, stage 3=93.6%, stage 4:91.8%, stage 5:88.1%	
	Ana Nunes, Jun, 2019[16]	Portugal	the Centro Hospitalar e Universitario de Coimbra:20 AD:28 PD:27 controls			OCT	support vector machine (SVM)	AD<br>PD	sensitivity<br>specificity<br>accuracy	sensitivity:AD:84.6%, PD:85.2%, HC:96.2%<br>specificity:AD:96.3%, PD:100%, HC:88.2%<br>accuracy:86.3%	
	Carlo Cavaliere, Dec. 2019[17]	Spain	Aragon—CEICA, Zaragoza, Spain:<br>48 cases:48 controls	leave-one-out cross-validation		OCT	SVM	MS	MCC<br>sensitivity<br>specificity<br>accuracy<br>AUC	MCC=0.81<br>sensitivity=0.89<br>specificity=0.92<br>accuracy=0.91<br>AUC=0.97	

	Sophie Lemmens,<br>Nov.2020[18]	Belgium	University Hospitals UZ Leuven:10 clinically probable AD or 7 biomarkerproven AD and 22controls	nested leave-one-out crossvalidation (LOOCV)		hyperspectral imaging,OCT:<br>1.S1 spectra<br>2.I2 spectra<br>3.S1 spectra+RNFL thickness<br>4.I2 spectra+RNFL thickness	Linear discriminant analysis (LDA) classifiers	AD	AUROC	1.AUROC=0.67<br>2.AUROC=0.70<br>3.AUROC=0.72<br>4.AUROC=0.79	
	Jianqiao Tian,<br>Jan.2021[19]	USA	UK Biobank:122 images of 87 AD:122 images of 87 controls--80%	--20%		CFP	U-Net<br>SVM-based classifier	AD	sensitivity<br>specificity<br>accuracy<br>F-1 score	sensitivity=0.7730-0.8418<br>specificity=0.8270-0.8664<br>accuracy=0.7999-0.8426<br>F-1 score=0.7942-0.8424	
	Alberto Montolío,Jun.2021[20]	Spain	Miguel Servet University Hospital:<br>108 cases:104 controls			OCT	multiple linear regression (MLR)<br>support vector machines (SVM)<br>decision tree (DT)<br>k-nearest neighbours (k-NN)<br>Naïve Bayes (NB)<br>ensemble classifier (EC)<br>long short-term m (LSTM) recurrent neural network	MS diagnosis<br>long-termprediction of MS disability course	accuracy<br>sensitivity<br>specificity<br>precision<br>AUC	MS diagnosis:EC:<br>accuracy: 87.7%<br>sensitivity: 87.0%<br>specificity: 88.5%<br>precision: 88.7%<br>AUC: 0.8775<br>long-termprediction of MS disability course:LSTM:<br>accuracy: 81.7%<br>sensitivity: 81.1%<br>specificity: 82.2%<br>precision: 78.9%<br>AUC: 0.8165	
	Qian Zhang,Nov.2021[21]	China	the Seventh Affiliated Hospital of Sun Yat-sen University:332 images of 86 subjects(22 dementia:26 MCI:38 controls)--58 subjects	--28 subjects		CFP	Support vector machine (SVM) and extreme learning machine (ELM)	dementia<br>MCI	sensitivity<br>specificity<br>AUC	SVM:<br>AUC=dementia:0.86,MCI:0.87,normal:0.85<br>ELM:<br>AUC=dementia:0.84,MCI:0.83,normal:0.81	
	Denis Corbin,<br>Apr.2022[22]	Canada	Canadian Longitudinal Study on Aging (CLSA):<br>18000 images	CLSA:<br>validation:3860 images<br>testing:3877 images		1.CFP<br>2.metadata<br>3.CFP+metadata	InceptionV3<br>MobilenetV2<br>EfficientNet	cognitive scores(global cognition,executive function, speed, memory and inhibition)	R^2<br>MAE	3.R^2=global cognition:0.224,executive function:0.156,speed:0.220,memory:0.035,inhibition:0.184	
	Carol Y. Cheung,<br>Aug.2022[23]	Singapore	National University Hospital and St. Luke's Hospital, Singapore:no cognitive impairment (NCI), cognitive impairment-no dementia (CIND), and dementia (n=491),5 years			1.CFP<br>2.CFP+age, gender,ethnicity and fellow calibre<br>3.CFP+education, cerebrovascular disease status, hypertension, hyperlipidemia, diabetes and smoking	SIVA-DLS	CRAE<br>CRVE	HR of cognitive decline<br>HR of dementia	HR of cognitive decline:<br>1.CRAE:1.198/SD,CRVE:0.989/SD<br>2.CRAE:1.341/SD,CRVE:1.281/SD<br>3.CRAE:1.258/SD,CRVE:1.204/SD<br>HR of dementia:<br>1.CRAE:1.364/SD,CRVE:0.815/SD<br>2.CRAE:1.443/SD,CRVE:1.227/SD<br>3.CRAE:1.624/SD,CRVE:1.460/SD	
	Xin Wang,Oct.2022[24]	China	Xiangya Hospital,the Third Xiangya Hospital:77 AD:145 controls--70%	--30%		OCTA	XGBoost<br>Light GBM<br>KNN<br>Random Forest<br>Gradient Boost<br>AdaBoost	AD	accuracy<br>AUC<br>f1 score<br>recall	accuracy=0.66-0.75<br>AUC=0.63-0.78<br>f1 score=0.52-0.72<br>recall=0.66-0.75	
**Neurodegeneration Diseases**	Carol Y Cheung,<br>Nov.2022[25]	Hong Kong, China	11 clinical studies and was done at 8 centres in 4 countries (Hong Kong Special Administrative Region, China, Singapore, the UK, and the USA):5598 images of 648 cases:7351 images of 3240 controls)			1.CFP<br>2.CFP+risk factors(ie, age, gender, and presence or absence of hypertension and diabetes;	EfficientNet-b2	Alzheimer's disease(AD)<br>amyloid β positive<br>AD+amyloid β positive	AUC<br>accuracy<br>sensitivity<br>specificity	AD:AUC=0.73-0.93<br>Amyloid β positive:AUC=0.68-0.86<br>AD+amyloid β positive:AUC=0.73-0.85	
	Xin Wang,Dec.2022[26]	China	Xiangya Hospital,the Third Xiangya Hospital:159 AD:299 controls--70%	--30%		OCT	extreme gradient boosting (XGBoost), Light Gradient Boosting Machine (Light GBM), k-nearest neighbor, Random Forest, Gradient Boost, and Adaptive Boosting (AdaBoost)	AD	accuracy<br>AUC<br>f1 score<br>recall	accuracy=0.70-0.74<br>AUC=0.68-0.75<br>f1 score=0.60-0.70<br>recall=0.70-0.74	
	Sangil Ahn,Mar.2023[27]	Republic of Korea	Kangbuk Samsung Hospital:615 subjects(539 images of 266 PD:700 images of 349 NON-PD)--rest	--80 subjects	Seoul National University Hospital and Yeungnam University Hospital	1.CFP<br>2.CFP+age,sex<br>3.CFP+sex,age,diabetes and hypertension data<br>4.CFP+sex,age,diabetes and hypertension data with multimodal method	ResNet-18	PD(H-Y scale and UPDRS-III score)	sensitivity<br>specificity<br>accuracy<br>AUROC	H-Y scale:<br>sensitivity=83.23%<br>specificity=66.81%<br>accuracy=73.38%<br>AUROC=0.77<br>UPDRS-III score:<br>sensitivity=82.61%<br>specificity=65.75%<br>accuracy=71.64%<br>AUROC=0.77	sensitivity=70.73%<br>specificity=66.66%<br>accuracy=70.45%<br>AUROC=0.67

	C. Ellis Wisely, Jun.2023[28]	USA	Duke Alzheimer’s Disease Research Center’s registry:<br>154 eyes of 80 cases:236 eyes of 129 controls<br>training:104 eyes:152 eyes<br>validation:20 eyes:24 eyes<br>testing:30 eyes:60 eyes			OCT,OCTA:<br>1.GC-IPL thickness maps<br>2.OCTA images<br>3.quantitative OCT and OCTA data<br>4.1+2<br>5.1+2+3	ResNet18	mild cognitive impairment(MCI)	AUC<br>accuracy<br>sensitivity<br>specificity	AUC=1:0.681,2:0.625,3:0.960,4:0.693,5:0.809	
	G. Luengnaruemitchai, Jul.2023[29]	Thailand	Chulalongkorn University:<br>81 images of 41 AD:66 images of 33 AD:78 images of 39 controls			CFP	MNetCDR<br>DenseNet-121	AD or MCI	accuracy<br>sensitivity<br>specificity<br>precision<br>F1 score	accuracy=96%<br>sensitivity=99%<br>specificity=90%<br>precision=95%<br>precision=97%	
	Hebei Gao,Sep.2023[30]	China	Daping Hospital, Army Medical University:<br>38 AD,29 MCI,50 controls<br>WMU Eye Hospital:<br>140 cases:133 controls			CFP+OCT	MFE modules<br>Cross-Modal-Fusion Unit	AD<br>MCI	accuracy<br>precision<br>recall<br>specificity<br>F1 score<br>AUC	accuracy=91.64%<br>AUC=96.78%	
	Mateo Gende, Nov.2023[31]	Spain	Miguel Servet Hospital:<br>1250 images of 50 patients(Alzheimer’s Disease:AD,Parkinson’s Disease:PD,Multiple Sclerosis:MS,Essential Tremor:ET,Healthy Control:HC)<br>--90%	--10%		OCT<br>(RNFL,GCL-BM)	MGU-Net	AD,PD,MS,ET,HC	precision<br>sensitivity<br>dice score<br>MAE	RNFL:<br>precision:0.850-0.946<br>sensitivity:0.910-0.958<br>dice score:0.882-0.943<br>GCL-BM:<br>precision:0.954-0.990<br>sensitivity:0.919-0.989<br>dice score:0.951-0.987	
	Je Moon Yoon, Jan.2024[32]	Republic of Korea	Samsung Medical Center:<br>85 scans of 55 subjects(31 scans of 20 AD:54 scans of 35 controls)	45 scans of 30 subjects(29 scans of 17 AD:16 scans of 13 controls)		OCTA+age,sex	1.ResNet,DenseNet, EfficientNet and Inception<br>2.LGBM<br>3.nnUNet,LGBM	AD	AUC<br>accuracy<br>sensitivity<br>specificity	AUC:<br>1:ResNet=50.6%,DenseNet=52.4%,EfficientNet=48%,Inception=51.2%<br>2:59.1%<br>2:72.2%	
	Rui Li,Jan.2024[33]	China	Kailuan community in Tangshan:908 subjects(491 cognitive impairment:417 controls)--80%	--20%		CFP	NFN+<br>Random Forest	cognitive decline	AUC	AUC=0.799	
**Psychiatric Diseases**	Abhishek Appaji, Mar.2022[34]	India	National Institute of Mental Health and Neurosciences (NIMHANS):198 subjects(116 cases:82 controls)	NIMHANS:<br>validation:56 subjects(33 cases:23 controls)<br>test:30 subjects(17 cases:13 controls)		CFP	CNN	schizophrenia	AUC	AUC=0.98	
	Charumathi Sabanayagam, Jun.2020[35]	Singapore	SEED(10376 images of 5188 patients,974 cases:4214 controls)	SEED(2594 images of 1297 patients,244 cases:1053 controls)	the Singapore Prospective Study Program(SP2)(7470 images of 3735 subjects,240 cases:3495 controls)<br>Beijing Eye Study(BES)(3076 images of 1538 subjects,53 cases:1485 controls)	A:CFP<br>B:risk factors including age,sex,ethnicity,diabetes,and hypertension<br>C:A+B	cCondenseNet	Chronic kidney disease(CKD)	AUC	A:AUC=0.911<br>B:AUC=0.916<br>C:AUC=0.938	SP2:AUC=A:0.733,B:0.829,C:0.810<br>BES:AUC=A:0.835,B:0.887,C:0.858
	Eugene Yu-Chuan Kang, Nov.2020[36]	Taiwan,China	Chang Gung Memorial Hospital(CGMH),Linkou Medical Center(20787 images of 4970 subjects)	Chang Gung Memorial Hospital(CGMH),Linkou Medical Center<br>validation:2189 images of 621 subjects<br>testing:2730 images of 621 subjects		CFP	VGG-19	early renal function impairment	AUC	AUC=0.81	
	Glen James, Jan.2021[37]	UK	UKB(117307 images of 59352 subjects)	UKB<br>validation:11837 images of 6427 subjects<br>test:6215 images of 3780 subjects		A:CFP<br>B:covariates(age, weight, sex, SBP, DBP, BMI, smoking, height, heart rate)<br>C:A+B	Inception v3	eGFR<br>current CKD<br>future CKD<br>future DM	MAE<br>AUC<br>R^2	eGFR:MAE=A:13.41,B:10.27,C:10.2mL/min/1.73m^2<br>current CKD:AUC=A:0.668,B:0.646,C:0.641<br>future CKD:AUC=0.633,B:0.627,C:0.651<br>future DM:AUC=0.749,B:0.748,C:0.732	
	Kang Zhang, Jun.2021[38]	China	China Consortium of Fundus Image Investigation cross-sectional dataset (CC-FII-C)(60244 images of 30122 subjects)	CC-FII-C:<br>tuning:8614 images from 4307 subjects<br>internal:17454 images of 8727 subjects	1.Guangdong province (16118 images of 8059 subjects)<br>2.China suboptimal health cohort study (COACS)(6162 images of 3081 subjects)	A:CFP<br>B:clinical metadata(age, sex, height, weight, body-mass index and blood pressure)<br>C:A+B	ResNet-50	CKD<br>early CKD<br>eGFR<br>incident CKD	AUC<br>R2<br>Pearson’s correlation coefficient (PCC)<br>HR	CKD:AUC=A:0.918,B:0.861,C:0.930<br>early CKD:AUC=A:0.839,B:0.805,C:0.864<br>eGFR:A:R^2=0.507,PCC=0.716,MAE=11.1 mL/min/1.73m^2<br>incident CKD:HR=1.87	CKD:1:AUC=A:0.885,B:0.842,C:0.898<br>CKD:2:AUC=A:0.870,B:0.817,C:0.897<br>early CKD:1:AUC=A:0.829,B:0.800,C:0.848<br>early CKD:2:AUC=A:0.834,B:0.787,C:0.845<br>eGFR:1:A:R^2=0.481,PCC=0.700,MAE=12.9mL/min/1.73m^2<br>eGFR:2:A:R^2=0.327,PCC=0.577,MAE=11.8mL/min/1.73m^2<br>incident CKD:HR=2.21

Renal Diseases	Shiran Zhang, May 2023[39]	China	UK biobank(19200 images of 11052 subjects)	UK biobank(35864 subjects)		1.CFP+age,sex,and ethnicity<br>2.1+deprivation, smoking status, drinking status, physical activity, diabetes mellitus, systolic blood pressure, body mass index, cholesterol, estimated glomerular filtration rate, and general health status	Xception	retinal age	HR of incident kidney failure	1.HR=1.09/year<br>2.HR=1.10/year	
	Young Su Joo, Jun.2023[40]	Republic of Korea	Severance Hospital(158216 images of 79108 subjects)	UKB(30447 subjects)<br>Korean Diabetic Cohort(5014 subjects)		CFP+clinical factors(age, sex, hypertension, diabetes)	ConvNeXT	Reti-CKD score	HR of CKD incidence	UK biobank:1.34/SD<br>Korean Diabetic Cohort:1.94/SD	
	Charumathi Sabanayagam, Sep.2023[41]	Singapore	SEED(1365 patients,162 cases:1203 controls)--80%	--20%		A:traditional risk factors<br>B:A+extended risk factors<br>C:B+imaging parameters<br>D:B+genetic parameters<br>E:B+blood metabolites<br>F:B+imaging parameters+blood metabolites+genetic parameters	logistic regression(LR)<br>least absolute shrinkage and selection operator(LASSO)<br>elastic net(EN)<br>classification and regression tree(CART)<br>random forest(RF)<br>gradient boosting decision tree(GBDT)<br>extreme gradient boosting(XGB)<br>support vector machine(SVM)<br>naïve Bayes(NB)	Incident DKD	AUC	for A-F:AUC=<br>LR:0.796,0.821,0.811,0.58,0.622,0.584<br>LASSO:0.781,0.811,0.806,0.773,0.814,0.772<br>EN:0.797,0.827,0.822,0.737,0.843,0.754<br>CART:0.702,0.744,0.742,0.666,0.703,0.631<br>RF:0.774,0.814,0.817,0.785,0.772,0.745<br>GBDT:0.789,0.807,0.807,0.783,0.809,0.779<br>XGB:0.764,0.801,0.804,0.77,0.801,0.788<br>SVM:0.604,0.744,0.75,0.722,0.728,0.723<br>NB:0.782,0.812,0.793,0.753,0.645,0.657	
	Yuliang Liu, Oct.2023[42]	China	Shandong Eye Hospital(35600 images of 66 subjects)--80%	--20%		OCT	3D ResNet architecture	diabetic nephropathy	accuracy, sensitivity and specificity	accuracy=91.68%<br>sensitivity=89.99%<br>specificity=92.18%	
	Shaomin Shi, Oct.2023[43]	China	Xiangyang Central Hospital(360 diabetes)	Xiangyang Central Hospital(155 diabetes)		1.age, gender, duration of diabetes, hypertension, history of cardiovascular and cerebrovascular disease, smoking, BMI(kg/m2), and glycosylated hemoglobin(%)<br>2.nonvascular area, total vessel tortuosity, total fractal dimension and vessel caliber from CFPs	RF<br>SVM<br>GBDT<br>AdaBoost	DKD	accuracy, sensitivity, specificity, F1 score, and AUC	accuracy=84.5%<br>sensitivity=84.5%<br>specificity=84.5%<br>F1 score=0.845<br>AUC=0.914	
	Bjorn Kaijun Betzler, Nov.2023[44]	Singapore	Singapore Integrated Diabetic Retinopathy Program(SiDRP)(13284 images of 6066 subjects,2556 cases:3510 controls)		1.SEED(3938 images of 1885 subjects,798 cases:1171 controls)<br>2.Singapore Macroangiopathy and Microvascular Reactivity in Type 2 Diabetes(SMART2D)(1424 images of 439 subjects,227 cases:485 controls)<br>3.Australian Eye and Heart Study(AHES)(460 diabetes)<br>4.Northern Ireland Cohort for the Longitudinal Study of Ageing (NICOLA)(265 diabetes)	A:CFP<br>B:risk factors adjusted for age,sex,ethnicity,diabetes duration,HbA1c,systolic blood pressure<br>C:A+B	ResNet18	diabetic kidney disease(DKD)	AUC	SiDRP:<br>AUC=A:0.826,B:0.847,C:0.866	SEED:AUC=A:0.764,B:0.802,C:0.828<br>SMART2D:AUC=A:0.726,B:0.701,C:0.761

	Songyang An, Nov.2023[45]	New Zealand	UK biobank(11232 images of 10325 subjects)	UK biobank(2808 images of 2750 subjects)		A:CFP(ResNet-50)<br>B:CFP(EfficientNetV2S)<br>C:CFP+metadata(age, gender, ethnicity)<br>D:Gaussian blur<br>E:Lightness channel contrast-limited histogram equalization (CLAHE)	ResNet-50<br>EfficientNetV2S	1:CKD with eGFR (creatinine only)<br>2:CKD with eGFR (serum creatinine and cystatin C)	AUC sensitivity specificity F1 scores	1:AUC=A:0.649,B:0.668,C:0.747,D:0.681,E:0.651<br>2:AUC=A:0.742,B:0.758,C:0.798,D:0.737,E:0.724	
Metabolic Diseases	Li Zhang, May 2020[46]	China	rural areas of Xinxiang County,Henan,in central China:1222 images of 625 subjects<br>training:--80%<br>tuning:--10%<br>testing:--10%			CFP	Inception-v3	hypertension<br>hyperglycemia<br>dyslipidemia<br>HCT<br>mean corpuscular hemoglobin concentration(MCHC)<br>acluster of CVD risk factors	AUC	hypertension:AUC=0.766<br>hyperglycemia:AUC=0.880<br>dyslipidemia:AUC=0.703	
	Jeremy Benson, Jul.2020[47]	USA	200 images of 46 cases:386 images of 96 controls<br>training:37 cases:77 controls<br>testing:8 cases:19 controls			CFP	VGG16	diabetic peripheral neuropathy (DPN)	accuracy<br>sensitivity<br>specificity	accuracy=89%<br>sensitivity=78%<br>specificity=95%	
	Yang Xiang, Apr.2021[48]	China	Ophthalmological Hospital of Tianjin Medical University:165 subjects			1.CFP<br>2.1+tongue and pulse conditions	RF	diabetes	accuracy<br>precision<br>recall<br>F1 scores	accuracy=1:0.53,2:0.85<br>precision=1:0.44,2:0.89<br>recall=1:0.39,2:0.67<br>F1 scores=1:0.41,2:0.76	
	Kang Zhang, Jun.2021[38]	China	China Consortium of Fundus Image Investigation cross-sectional dataset (CC-FII-C)(60244 images of 30122 subjects)	CC-FII-C:<br>tuning:8614 images from 4307 subjects<br>internal:17454 images of 8727 subjects	1.Guangdong province (16118 images of 8059 subjects)<br>2.China suboptimal health cohort study (COACS)(6162 images of 3081 subjects)	A:CFP<br>B:clinical metadata(age, sex, height, weight, body-mass index and blood pressure)<br>C:A+B	ResNet-50	T2DM<br>incident T2DM	AUC<br>R^2<br>Pearson's correlation coefficient (PCC)<br>HR	T2DM:AUC=A:0.923,B:0.828,C:0.929<br>incident T2DM:HR:1.46	T2DM:1:AUC=A:0.854,B:0.796,C:0.871<br>T2DM:2:AUC=A:0.820,B:0.762,C:0.845<br>incident T2DM:HR:1.90
	Jae-Seung Yun, May 2022[49]	USA	UKB(69639 images of 37904 subjects)	UKB(tuning:22342 images of 12173 subjects,validation:22394 images of 12185 subjects)		1.CFP<br>2.traditional risk factors(TRFs)<br>3.1+2	ResNet18	type 2 diabetes and risk factors	R^2<br>AUC<br>sensitivity<br>specificity, PPV<br>NPV	type 2 diabetes:<br>AUC=1:0.731,2:0.810,3:0.844	
	Zhuoting Zhu, Mar.2023[50]	China	UK biobank(19200 images of 11052 subjects)	test:<br>UKB(35918 subjects)		CFP	Xception	retinal age gap	OR of:<br>central obesity<br>hypertension<br>dyslipidemia<br>hypertriglyceridemia<br>hyperglycemia<br>high-sensitivity C-reactive protein	OR:<br>Metabolic syndrome:Q2:1.05,Q3:1.10,Q4:1.14<br>Inflammation:Q2:1.02,Q3:1.10,Q4:1.25<br>Combined:Q2:1.04,Q3:1.11,Q4:1.20	
	Hamada R. H. Al-Absi, Jan.2024[51]	Qatar	Total: 15011 images of 5545 subjects (7515 images of 2540 cases:7496 images of 3005 controls)<br>Qatar Biobank(QBB):2099 cases:2806 controls<br>Hamad Medical Corporation(HMC):883 images of 442 cases:396 images of 199 controls			CFP	DenseNet-121<br>ResNet-50<br>EfficientNet<br>VGG-11<br>MobileNet_v2	diabetes	accuracy<br>sensitivity<br>specificity<br>precision<br>F1-score<br>MCC	VGG-11:<br>accuracy=92.63%<br>sensitivity=93.93%<br>specificity=91.32%<br>precision=91.76%<br>F1-score=92.81%<br>MCC=85.32%	
Hepatobiliary Diseases	Wei Xiao, Feb.2021[52]	China	the Third Affiliated Hospital of Sun Yat-sen University, the Affiliated Huadu Hospital of Southern Medical University, and the Nantian Medical Centre of Aikang Health Care [Guangzhou, China](1989 images of 1138 subjects,718 cases:420 controls)	the Third Affiliated Hospital of Sun Yatsen University and the Huanshidong Medical Centre of Aikang Health Care [Guangzhou, China])(800 images of 468 subjects,220 cases:248 controls)		CFP	ResNet-101<br>VGG-16<br>EfficientNet-B0	hepatobiliary diseases<br>liver cancer<br>liver cirrhosis<br>chronic viral hepatitis<br>non-alcoholic fatty liver disease<br>cholelithiasis<br>hepatic cyst	AUROC,sensitivity,specificity,and F1* score	screening:AUROC=0.68<br>identification:<br>liver cancer:AUROC=0.84<br>liver cirrhosis:AUROC=0.83<br>chronic viral hepatitis:AUROC=0.62<br>non-alcoholic fatty liver disease:AUROC=0.70<br>cholelithiasis:AUROC=0.68<br>hepatic cyst:AUROC=0.69	

**Anaemia**	Zailiang Chen, Apr.2019[53]	China	the Second Xiangya Hospital of Central South University (316 images of 24 patients; 255 images of 16 normal subjects)--39 subjects	remaining 1 subject		OCT	linear discriminant analysis (DA) classifier<br>decision tree (DT) classifier<br>k-nearest neighbor (KNN) classifier<br>naive Bayes (NB) classifier<br>support vector machine (SVM)	anaemia	accuracy	DA:accuracy=0.8358<br>SVM:accuracy=0.7839<br>DT:accuracy=0.7094<br>NB:accuracy=0.6716<br>KNN:accuracy=0.6625	
	Akinori Mitani, Jan.2020[54]	USA	training: UK Biobank (80006 images of 40041 subjects)<br>tuning: UK Biobank (11457 images of 5734 subjects)	UK Biobank(UKB)(22742 images of 11388 subjects)	self-reported diabetes (539 participants)	A:CFP<br>B:metadata(race or ethnicity, age, sex and blood pressure)<br>C:A+B	Inception-v4 architecture	haemoglobin concentration(Hb), haematocrit (HCT) and red blood cell count (RBC), anaemia, moderate anaemia	MAE, AUC	Hb:MAE=A:0.67, B:0.73, C:0.63g/dl<br>Hb:AUC=A:0.87, B:0.74, C:0.88<br>anaemia:AUC=A:0.87, B:0.73, C:0.88<br>moderate anaemia:AUC=A:0.95, B:0.79, C:0.95	MAE=C:0.73g/dl<br>anaemia:AUC=C:0.89
	Tyler Hyungtaek Rim, Oct.2020[55]	Singapore	Severance Main Hospital(86994 images of 43497 subjects)	Severance Main Hospital(21698 images of 10849 subjects)	Severance Gangnam Hospital(9324 images of 4662 subjects), The Singapore Epidemiology of Eye Diseases(SEED) study(63275 images of 7726 subjects), UKB(50732 images of 25366 subjects)	CFP	VGG16	HCT, Hb, RBC	MAE<br>R^2	HCT:MAE=2.03%, R^2=0.57<br>Hb:MAE=0.79g/dl, R^2=0.56<br>RBC:MAE=0.26*10^{12}/L, R^2=0.45	Severance Gangnam Hospital:<br>HCT:MAE=2.81%, R^2=0.26<br>Hb:MAE=0.96g/dl, R^2=0.33<br>RBC:MAE=0.35*10^{12}/L, R^2=0.14<br>SEED:<br>Hb:MAE=0.98g/dl, R^2=0.32<br>RBC:MAE=0.37*10^{12}/L, R^2=0.14<br>UKB:<br>HCT:MAE=2.62%, R^2=0.09<br>Hb:MAE=0.93g/dl, R^2=0.06<br>RBC:MAE=0.33*10^{12}/L, R^2=-0.02
	Hao Wei, Apr.2021[56]	China	the Second Xiangya Hospital of Central South University (221 images of 17 patients; 207 images of 13 normal subjects)--randomly 80%	rest 20%		OCT	VGG-11//16<br>Resnet//16<br>ShuffleNetV1<br>ShuffleNetV2<br>SENet//16<br>AneNet	anaemia	accuracy, sensitivity, and specificity, AUC	AneNet:<br>accuracy=0.9865, sensitivity=0.9838, specificity=0.9594, AUC=0.9983	
	Xinyu Zhao, May 2022[57]	China	Peking Union Medical College Hospital (9221 images of 2445 subjects)	Peking Union Medical College Hospital (validation: 577 images of 213 subjects; test: 1730 images of 565 subjects)		UWF images	InceptionResNetV2	Hb, anaemia	MAE, AUC	Hb:MAE=0.83g/dl<br>anaemia:AUC=0.93	
**Fibromyalgia**	Luciano Boquete, May.2022[58]	Spain	Miguel Servet Hospital:<br>29 FM:32 controls			OCT	RUSBoosted tree classifier	fibromyalgia	accuracy<br>AUC	accuracy=0.82<br>AUC=0.82	

References

1. Gerrits N, Elen B, Craenendonck TV, Triantafyllidou D, Petropoulos IN, Malik RA, et al. Age and sex affect deep learning prediction of cardiometabolic risk factors from retinal images. Sci Rep. 2020; 10: 9432.
2. Rim TH, Lee CJ, Tham YC, Cheung N, Yu M, Lee G, et al. Deep-learning-based cardiovascular risk stratification using coronary artery calcium scores predicted from retinal photographs. Lancet Digit Health. 2021; 3: e306-e16.
3. Cheung CY, Xu D, Cheng CY, Sabanayagam C, Tham YC, Yu M, et al. A deep-learning system for the assessment of cardiovascular disease risk via the measurement of retinal-vessel calibre. Nat Biomed Eng. 2021; 5: 498-508.
4. Al-Absi HRH, Islam MT, Refaee MA, Chowdhury MEH, Alam T. Cardiovascular Disease Diagnosis from DXA Scan and Retinal Images Using Deep Learning. Sensors (Basel). 2022; 22: 4310.
5. Rudnicka AR, Welikala R, Barman S, Foster PJ, Luben R, Hayat S, et al. Artificial intelligence-enabled retinal vasculometry for prediction of circulatory mortality, myocardial infarction and stroke. Br J Ophthalmol. 2022; 106: 1722-9.
6. Lui G, Leung HS, Lee J, Wong CK, Li X, Ho M, et al. An efficient approach to estimate the risk of coronary artery disease for people living with HIV using machine-learning-based retinal image analysis. PLoS One. 2023; 18: e0281701.
7. Mellor J, Jiang W, Fleming A, McGurnaghan SJ, Blackbourn L, Styles C, et al. Can deep learning on retinal images augment known risk factors for cardiovascular disease prediction in diabetes? A prospective cohort study from the national screening programme in Scotland. Int J Med Inform. 2023; 175: 105072.
8. Lim CC, Chong C, Tan G, Tan CS, Cheung CY, Wong TY, et al. A deep learning system for retinal vessel calibre improves cardiovascular risk prediction in Asians with chronic kidney disease. Clin Kidney J. 2023; 16: 2693-702.
9. Qu Y, Zhuo Y, Lee J, Huang X, Yang Z, Yu H, et al. Ischemic and haemorrhagic stroke risk estimation using a machine-learning-based retinal image analysis. Front Neurol. 2022; 13: 916966.
10. Zhu Z, Hu W, Chen R, Xiong R, Wang W, Shang X, et al. Retinal age gap as a predictive biomarker of stroke risk. BMC Med. 2022; 20: 466.
11. Pachade S, Coronado I, Abdelkhaleq R, Yan J, Salazar-Marioni S, Jagolino A, et al. Detection of Stroke with Retinal Microvascular Density and Self-Supervised Learning Using OCT-A and Fundus Imaging. J Clin Med. 2022; 11: 7408.
12. Shu L, Zhong K, Chen N, Gu W, Shang W, Liang J, et al. Predicting the severity of white matter lesions among patients with cerebrovascular risk factors based on retinal images and clinical laboratory data: a deep learning study. Front Neurol. 2023; 14: 1168836.

13. Li H, Gao M, Song H, Wu X, Li G, Cui Y, et al. Predicting ischemic stroke risk from atrial fibrillation based on multi-spectral fundus images using deep learning. Front Cardiovasc Med. 2023; 10: 1185890.
14. An L, Qin J, Jiang W, Luo P, Luo X, Lai Y, et al. Non-invasive and accurate risk evaluation of cerebrovascular disease using retinal fundus photo based on deep learning. Front Neurol. 2023; 14: 1257388.
15. Hong J, Yoon S, Shim KW, Park YR. Screening of Moyamoya Disease From Retinal Photographs: Development and Validation of Deep Learning Algorithms. Stroke. 2024; 55: 715-24.
16. Nunes A, Silva G, Duque C, Januário C, Santana I, Ambrósio AF, et al. Retinal texture biomarkers may help to discriminate between Alzheimer's, Parkinson's, and healthy controls. PLoS One. 2019; 14: e0218826.
17. Cavaliere C, Vilades E, Alonso-Rodríguez MC, Rodrigo MJ, Pablo LE, Miguel JM, et al. Computer-Aided Diagnosis of Multiple Sclerosis Using a Support Vector Machine and Optical Coherence Tomography Features. Sensors (Basel). 2019; 19: 5323.
18. Lemmens S, Van Craenendonck T, Van Eijgen J, De Groef L, Bruffaerts R, de Jesus DA, et al. Combination of snapshot hyperspectral retinal imaging and optical coherence tomography to identify Alzheimer's disease patients. Alzheimers Res Ther. 2020; 12: 144.
19. Tian J, Smith G, Guo H, Liu B, Pan Z, Wang Z, et al. Modular machine learning for Alzheimer's disease classification from retinal vasculature. Sci Rep. 2021; 11: 238.
20. Montolío A, Martín-Gallego A, Cegoñino J, Orduna E, Vilades E, Garcia-Martin E, et al. Machine learning in diagnosis and disability prediction of multiple sclerosis using optical coherence tomography. Comput Biol Med. 2021; 133: 104416.
21. Zhang Q, Li J, Bian M, He Q, Shen Y, Lan Y, et al. Retinal Imaging Techniques Based on Machine Learning Models in Recognition and Prediction of Mild Cognitive Impairment. Neuropsychiatr Dis Treat. 2021; 17: 3267-81.
22. Corbin D, Lesage F. Assessment of the predictive potential of cognitive scores from retinal images and retinal fundus metadata via deep learning using the CLSA database. Sci Rep. 2022; 12: 5767.
23. Cheung CY, Wong WLE, Hilal S, Kan CN, Gyanwali B, Tham YC, et al. Deep-learning retinal vessel calibre measurements and risk of cognitive decline and dementia. Brain Commun. 2022; 4: fcac212.
24. Wang X, Wang Y, Liu H, Zhu X, Hao X, Zhu Y, et al. Macular Microvascular Density as a Diagnostic Biomarker for Alzheimer's Disease. J Alzheimers Dis. 2022; 90: 139-49.
25. Cheung CY, Ran AR, Wang S, Chan VTT, Sham K, Hilal S, et al. A deep learning model for detection of Alzheimer's disease based on retinal photographs: a retrospective, multicentre case-control study. Lancet Digit Health. 2022; 4: e806-e15.

26. Wang X, Jiao B, Liu H, Wang Y, Hao X, Zhu Y, et al. Machine learning based on Optical Coherence Tomography images as a diagnostic tool for Alzheimer's disease. CNS Neurosci Ther. 2022; 28: 2206-17.
27. Ahn S, Shin J, Song SJ, Yoon WT, Sagong M, Jeong A, et al. Neurologic Dysfunction Assessment in Parkinson Disease Based on Fundus Photographs Using Deep Learning. JAMA Ophthalmol. 2023; 141: 234-40.
28. Wisely CE, Richardson A, Henao R, Robbins CB, Ma JP, Wang D, et al. A Convolutional Neural Network Using Multimodal Retinal Imaging for Differentiation of Mild Cognitive Impairment from Normal Cognition. Ophthalmol Sci. 2024; 4: 100355.
29. Luengnaruemitchai G, Kaewmahanin W, Munthuli A, Phienphanich P, Puangarom S, Sangchocanonta S, et al. Alzheimer's Together with Mild Cognitive Impairment Screening Using Polar Transformation of Middle Zone of Fundus Images Based Deep Learning. Annu Int Conf IEEE Eng Med Biol Soc. 2023; 2023: 1-4.
30. Gao H, Zhao S, Zheng G, Wang X, Zhao R, Pan Z, et al. Using a dual-stream attention neural network to characterize mild cognitive impairment based on retinal images. Comput Biol Med. 2023; 166: 107411.
31. Gende M, Mallen V, de Moura J, Cordon B, Garcia-Martin E, Sanchez CI, et al. Automatic Segmentation of Retinal Layers in Multiple Neurodegenerative Disorder Scenarios. IEEE J Biomed Health Inform. 2023; 27: 5483-94.
32. Yoon JM, Lim CY, Noh H, Nam SW, Jun SY, Kim MJ, et al. Enhancing foveal avascular zone analysis for Alzheimer's diagnosis with AI segmentation and machine learning using multiple radiomic features. Sci Rep. 2024; 14: 1841.
33. Li R, Hui Y, Zhang X, Zhang S, Lv B, Ni Y, et al. Ocular biomarkers of cognitive decline based on deep-learning retinal vessel segmentation. BMC Geriatr. 2024; 24: 28.
34. Appaji A, Harish V, Korann V, Devi P, Jacob A, Padmanabha A, et al. Deep learning model using retinal vascular images for classifying schizophrenia. Schizophr Res. 2022; 241: 238-43.
35. Sabanayagam C, Xu D, Ting DSW, Nusinovici S, Banu R, Hamzah H, et al. A deep learning algorithm to detect chronic kidney disease from retinal photographs in community-based populations. Lancet Digit Health. 2020; 2: e295-e302.
36. Kang EY, Hsieh YT, Li CH, Huang YJ, Kuo CF, Kang JH, et al. Deep Learning-Based Detection of Early Renal Function Impairment Using Retinal Fundus Images: Model Development and Validation. JMIR Med Inform. 2020; 8: e23472.
37. James G, Mohankumar, G., Cooper, A.J., Siddiqui, A., Fenici, P., Markham, L., et al. T. Predicting Renal Disease and Associated Complications Through Deep Learning Using Retinal Fundus Images Linked to Clinical Data. SSRN Electronic Journal. 2021: n.pag.

38. Zhang K, Liu X, Xu J, Yuan J, Cai W, Chen T, et al. Deep-learning models for the detection and incidence prediction of chronic kidney disease and type 2 diabetes from retinal fundus images. Nat Biomed Eng. 2021; 5: 533-45.
39. Zhang S, Chen R, Wang Y, Hu W, Kiburg KV, Zhang J, et al. Association of Retinal Age Gap and Risk of Kidney Failure: A UK Biobank Study. Am J Kidney Dis. 2023; 81: 537-44.e1.
40. Joo YS, Rim TH, Koh HB, Yi J, Kim H, Lee G, et al. Non-invasive chronic kidney disease risk stratification tool derived from retina-based deep learning and clinical factors. NPJ Digit Med. 2023; 6: 114.
41. Sabanayagam C, He F, Nusinovici S, Li J, Lim C, Tan G, et al. Prediction of diabetic kidney disease risk using machine learning models: A population-based cohort study of Asian adults. Elife. 2023; 12: e81878.
42. Liu Y, Zhang F, Gao X, Liu T, Dong J. Lesion-aware attention network for diabetic nephropathy diagnosis with optical coherence tomography images. Front Med (Lausanne). 2023; 10: 1259478.
43. Shi S, Gao L, Zhang J, Zhang B, Xiao J, Xu W, et al. The automatic detection of diabetic kidney disease from retinal vascular parameters combined with clinical variables using artificial intelligence in type-2 diabetes patients. BMC Med Inform Decis Mak. 2023; 23: 241.
44. Betzler BK, Chee EYL, He F, Lim CC, Ho J, Hamzah H, et al. Deep learning algorithms to detect diabetic kidney disease from retinal photographs in multiethnic populations with diabetes. J Am Med Inform Assoc. 2023; 30: 1904-14.
45. An S, Vaghefi E, Yang S, Xie L, Squirrell D. Examination of alternative eGFR definitions on the performance of deep learning models for detection of chronic kidney disease from fundus photographs. PLoS One. 2023; 18: e0295073.
46. Zhang L, Yuan M, An Z, Zhao X, Wu H, Li H, et al. Prediction of hypertension, hyperglycemia and dyslipidemia from retinal fundus photographs via deep learning: A cross-sectional study of chronic diseases in central China. PLoS One. 2020; 15: e0233166.
47. Benson J, Estrada T, Burge M, Soliz P. Diabetic Peripheral Neuropathy Risk Assessment using Digital Fundus Photographs and Machine Learning. Annu Int Conf IEEE Eng Med Biol Soc. 2020; 2020: 1988-91.
48. Xiang Y, Shujin L, Hongfang C, Yinping W, Dawei Y, Zhou D, et al. Artificial Intelligence-Based Diagnosis of Diabetes Mellitus: Combining Fundus Photography with Traditional Chinese Medicine Diagnostic Methodology. Biomed Res Int. 2021; 2021: 5556057.
49. Yun JS, Kim J, Jung SH, Cha SA, Ko SH, Ahn YB, et al. A deep learning model for screening type 2 diabetes from retinal photographs. Nutr Metab Cardiovasc Dis. 2022; 32: 1218-26.
50. Zhu Z, Liu D, Chen R, Hu W, Liao H, Kiburg K, et al. The Association of Retinal age gap with metabolic syndrome and inflammation. J Diabetes. 2023; 15: 237-45.

51. Al-Absi HRH, Pai A, Naeem U, Mohamed FK, Arya S, Sbeit RA, et al. DiaNet v2 deep learning based method for diabetes diagnosis using retinal images. Sci Rep. 2024; 14: 1595.
52. Xiao W, Huang X, Wang JH, Lin DR, Zhu Y, Chen C, et al. Screening and identifying hepatobiliary diseases through deep learning using ocular images: a prospective, multicentre study. Lancet Digit Health. 2021; 3: e88-e97.
53. Chen Z, Mo Y, Ouyang P, Shen H, Li D, Zhao R. Retinal vessel optical coherence tomography images for anemia screening. Med Biol Eng Comput. 2019; 57: 953-66.
54. Mitani A, Huang A, Venugopalan S, Corrado GS, Peng L, Webster DR, et al. Detection of anaemia from retinal fundus images via deep learning. Nat Biomed Eng. 2020; 4: 18-27.
55. Rim TH, Lee G, Kim Y, Tham YC, Lee CJ, Baik SJ, et al. Prediction of systemic biomarkers from retinal photographs: development and validation of deep-learning algorithms. Lancet Digit Health. 2020; 2: e526-e36.
56. Wei H, Shen H, Li J, Zhao R, Chen Z. AneNet: A lightweight network for the real-time anemia screening from retinal vessel optical coherence tomography images. Optics & Laser Technology. 2021; 136: 106773.
57. Zhao X, Meng L, Su H, Lv B, Lv C, Xie G, et al. Deep-Learning-Based Hemoglobin Concentration Prediction and Anemia Screening Using Ultra-Wide Field Fundus Images. Front Cell Dev Biol. 2022; 10: 888268.
58. Boquete L, Vicente MJ, Miguel-Jiménez JM, Sánchez-Morla EM, Ortiz M, Satue M, et al. Objective Diagnosis of Fibromyalgia Using Neuroretinal Evaluation and Artificial Intelligence. Int J Clin Health Psychol. 2022; 22: 100294.
